# Fear Avoidance Beliefs and Risk of Long-Term Sickness Absence: Prospective Cohort Study among Workers with Musculoskeletal Pain

**DOI:** 10.1155/2018/8347120

**Published:** 2018-09-02

**Authors:** Kenneth Jay, Sannie Vester Thorsen, Emil Sundstrup, Ramon Aiguadé, Jose Casaña, Joaquin Calatayud, Lars Louis Andersen

**Affiliations:** ^1^National Research Centre for the Working Environment, Lersø Parkallé 105, Copenhagen, Denmark; ^2^The Carrick Institute for Graduate studies, Institute of Clinical Neuroscience and Rehabilitation, 8910 Astronaut Blvd, Cape Canaveral, LF 32920, USA; ^3^Departamento de Enfermería y Fisioterapia, Universidad de Lleida, Cataluña, Spain; ^4^Exercise Intervention for Health Research Group (EXINH-RG), Department of Physiotherapy, University of Valencia, Spain; ^5^Sport Sciences, Department of Health Science and Technology, Aalborg University, Denmark

## Abstract

**Background and Objective:**

Musculoskeletal pain is common in the population. Negative beliefs about musculoskeletal pain and physical activity may lead to avoidance behavior resulting in absence from work. The present study investigates the influence of fear avoidance beliefs on long-term sickness absence.

**Methods:**

Workers of the general working population with musculoskeletal pain (low back, neck/shoulder, and/or arm/hand pain; n = 8319) from the Danish Work Environment Cohort Study were included. Long-term sickness absence data were obtained from the Danish Register for Evaluation and Marginalization (DREAM). Time-to-event analyses (cox regression) controlled for various confounders estimated the association between fear avoidance beliefs (very low, low, moderate [reference category], high, and very high) at baseline and long-term sickness absence (LTSA; ≥6 consecutive weeks) during a 2-year follow-up.

**Results:**

During the 2-year follow-up, 10.2% of the workers experienced long-term sickness absence. In the fully adjusted model, very high-level fear avoidance increased the risk of LTSA with hazard ratio (HR) of 1.48 (95% CI 1.15-1.90). Similar results were seen analyses stratified for occupational physical activity, i.e., sedentary workers (HR 1.72 (95% CI 1.04-2.83)) and physically active workers (HR 1.48 (95% CI 1.10-2.01)).

**Conclusion:**

A very high level of fear avoidance is a risk factor for long-term sickness absence among workers with musculoskeletal pain regardless of the level of occupational physical activity. Future interventions should target fear avoidance beliefs through information and campaigns about the benefits of staying active when having musculoskeletal pain.

## 1. Introduction

The Fifth European Working Condition Survey from 2010 shows that sickness absence is a challenge in all the Nordic countries [1]. It is estimated that employers' costs for wages of absent employees amount to about DKK 25 billion (€3.36 billion) a year, not considering the enterprises' contributions to sickness absence insurance.

Chronic musculoskeletal pain is a major cause of sickness absence [2]. However, biology alone cannot explain the occurrence of chronic pain and sickness absence, and psychological and social factors must also be considered [3]. For example, thoughts and beliefs about pain and movement may be important in the interplay between pain and the decision for a worker to either call in sick or go to work. Explained by Vlaeyen & Linton, the fear avoidance model of chronic pain states that the pain experience may in some individuals start a process of negative affect and catastrophizing thoughts that lead to defensive motivation, elevated arousal, or feeling threatened due to fear of experiencing pain [4, 5]. Thinking of the fear avoidance model in relation to work, a high level of fear avoidance can be speculated to lead to avoidance behavior in relation to work activities and thus increased risk of absence from work. Contrastingly, when pain is not considered a threat, the individual will likely remain physically active and engaged in everyday activities and therefore also work [6].

The degree of fear avoidance beliefs (FAB) is commonly assessed by the two-part Fear Avoidance Beliefs Questionnaire (FABQ) where part one focuses on fear avoidance in relation to leisure-time activities (e.g., bending, lifting, walking, or driving) and part two focuses on physical activities related to the individual's work [7]. In patients with chronic back pain, high levels of fear avoidance have been shown to increase the risk of poor work-related outcomes such as sick leave and not returning to work [8]. Fear avoidance beliefs can therefore be construed as mediators between pain and avoidance behaviors, such as sick leave, early retirement, or withdrawal from working life [9, 10]. Thus, we hypothesize that a high level of fear avoidance increases the risk of sick leave, because the worker fears that working will aggravate the pain situation and will thus be more likely to stay at home and rest.

The aim of this study is to investigate the impact of fear avoidance beliefs on register-based long-term sickness absence in the general working population.

## 2. Methods

### 2.1. Study Design

The present study uses data from the 2010 round of the Danish Work Environment Cohort Study (DWECS) a with 2-year follow-up from the Danish Register for Evaluation and Marginalization (DREAM). The DWECS consists of questionnaires assessing work environment and health in the Danish general working population. The DREAM register holds information about all social transfer payments for all citizens in Denmark, including granted sickness absence compensation. In this study, long-term sickness absence is defined as receiving sickness absence compensation of 6 weeks or more according to DREAM.

### 2.2. Participants

The questionnaire was distributed to a random sample drawn from the Danish Central Population Register of 30,000 people between 18 and 59 years with residence in Denmark, of which two-thirds were from the working population [11]. A total of 10,605 (~53%) from the working population replied to the 2010 questionnaire. For the present analyses, we only included currently employed wage earners with musculoskeletal pain (>= 1 scale 0-9 in the low back, neck/shoulder, and/or arm/hand; n = 8319). Characteristics of the study population are shown in Table 1.

### 2.3. Registration and Ethical Approval

The Danish Data Protection Agency (Datatilsynet, Denmark) has registered and journalized the DWECS (journal number 2007-54-0059). All data were deidentified and analyzed anonymously. According to Danish legislation, questionnaire- and register-based studies do not need approval by ethical and scientific committees nor informed consent.

### 2.4. Explanatory Variable: Fear Avoidance Beliefs

Two questions about fear avoidance beliefs from the Fear Avoidance Beliefs Questionnaire were included in the DWECS questionnaire survey. The first question was selected from the physical activity subscale (FABQPA): “Physical activity makes my pain worse”; the second question was selected from the work subscale (FABQW): “I should not do my normal work with my present pain.” Participants replied on a 7-point Likert scale from completely agree to disagree. For further analyses, the responses were averaged and normalized to a scale of 0 to 100 (higher number equals higher level of disagreement) and further subdivided in 5 categories with the following score ranges: (1) “*very low fear avoidance*” [from 0 to 20], (2) “*low fear avoidance*” [from 20-40], “*moderate fear avoidance*” [from 40 to 60], “*high fear avoidance*” [from 60 to 80], and “*very high fear avoidance*” [from 80 to 100].

### 2.5. Outcome Variable: Long-Term Sickness Absence

Information about long-term sickness absence was obtained from the Danish Register for Evaluation and Marginalization (DREAM) and linked to DWECS by the individual social security number assigned to all Danish residents at birth or immigration. DREAM contains information on who received social transfer payments since 1991, e.g., sickness absence compensation, disability pension, and early retirement. Sickness absence compensation is given to the employer, who can apply for a refund from the state for employees after 30 calendar days of sickness absence. LTSA was defined as having at least 6 consecutive weeks in DREAM [12–14].

### 2.6. Control Variables

Control variables were added in two statistical models. Model 1 was a minimally adjusted model, adjusting only for age and gender. Model 2 was the fully adjusted model, adjusting for age, gender, job group (86 categories), psychosocial work environment (influence at work, emotional demands, support from colleagues, and support from leader) measured by the scales developed to COPSOQ [15], physical work demands (4 categories from sedentary to hard physical work), lifestyle (smoking, leisure physical activity, BMI), musculoskeletal pain intensity (low back, neck/shoulder, and arm/hand), previous long-term sickness absence (over the preceding two years prior to baseline), mental health, and chronic disease (depression, diabetes, cardiovascular disease, asthma, cancer, and back disease) [16].

### 2.7. Statistical Methods

The Cox proportional hazard models (Proc PHREG, SAS version 9.4) were applied in modelling the prospective association between fear avoidance and risk of LTSA. All events of permanent labor market drop out based on the DREAM register (i.e., statutory retirement age, early retirement, disability pension, immigration, or death) were censored at the time they occurred during the two-year follow-up. When LTSA occurred, the survival times were noncensored and referred to as event times. In addition to the main analysis, we also performed analyses stratified for occupational physical activity (1) sedentary workers and (2) physically active workers (3 subgroups combined). Proportional hazard assumptions were tested by visual inspection. Results are reported as hazard ratios (HR) with 95% confidence intervals (CI).

## 3. Results

Table 1 shows that there were slightly more women than men in the sample. Approximately half were nonsmokers and a quarter current smokers. Regarding physical activities most had a moderate level during leisure and about one-half had sedentary work and the other half had physically active work, respectively. Average pain intensity was about 3 in the neck/shoulder and low back and less than 2 in the arm/hand. 9.4% had experienced LTSA during the 2 years prior to baseline. During the 2-year follow-up, 10.2% experienced LTSA (8.1% among sedentary workers, 11.8% among physically active workers).

Table 2 shows the estimates for the minimally and fully adjusted models, as well as the analyses stratified for occupational physical activity. Moderate fear avoidance beliefs were set as reference (value = 1). In the minimally adjusted model (model 1) for all workers, very low fear avoidance was associated with reduced risk of LTSA (HR 0.76) and high (HR 1.31) and very high (HR 1.80) with increased risk. However, in the fully adjusted model (model 2), only a very high-level fear avoidance increased the risk of LTSA with hazard ratio (HR) of 1.48 (95% CI 1.15-1.90). In terms of understanding the size of this effect, a hazard ratio of 1.48 equals an increased risk of 48% ((HR-1)*∗*100) for long-term sickness absence in the “very high fear avoidance” compared with the “moderate fear avoidance” group. Thus, it can be considered quite a large effect. Similar results were seen analyses stratified for occupational physical activity, i.e., sedentary workers (HR 1.72 (95% CI 1.04-2.83)) and physically active workers (HR 1.48 (95% CI 1.10-2.01)), i.e., increased risks of 72% and 48%, respectively, in the “very high fear avoidance” compared with the “moderate fear avoidance” group.

## 4. Discussion

The main finding of this study is that a very high level of fear avoidance is a risk factor for long-term sickness absence even when adjusting to various confounders. This was seen regardless of the level of occupational physical activity, and fear avoidance can therefore be considered as a general risk factor for absence from work. Below we will discuss the present findings in relation to (1) possible underlying causes of the link between fear avoidance and sickness absence and (2) possible targets for action at the workplaces.

The present analysis showed a hazard ratio of 1.48 in fully adjusted model and 1.80 in the minimally adjusted model, corresponding to 48% and 80% increased risk in the “very high FAB” group compared with “moderate FAB group.” Thus, the analyses showed that very high FAB is a risk factor for sickness absence even when adjusted to pain intensity and the physical demands in the work. Thus, FAB may not only be a mediator between the physical and psychosocial work conditions and risk of long-term sickness absence but also an independent explanatory variable. FAB is a complex phenomenon, shaped in the interplay between internal and external stressors, from competing personal goals, psychosocial factors, and daily life and workplace factors [9]. Our results fit well with the fear avoidance model; i.e., the workers with high fear avoidance are, according to the definition of fear avoidance, more likely to feel threatened due to fear of experiencing pain when moving the body [4, 5]. In this sense, work can be considered a behavior that the workers with high fear avoidance are more likely to avoid, i.e., more likely of calling in sick. In the long-term this disuse behavior may induce a vicious cycle that is difficult to break, i.e., leading to long-term sickness absence.

In relation to return to work after long-term sickness absence it may be beneficial to address FAB directly in workplace health promotion programs. For instance, Fritz & George [17] found by using the work subscale of the FABQ in patients with acute work-related low back pain that work-related FAB is the strongest predictor of return to work among the psychosocial risk factors [17].

Although the present study was not an intervention, the results suggest that future intervention targeting a reduction in FAB in the working population may have the potential to reduce sickness absence. Multifactorial intervention strategies focusing on physical activity as well as targeted physical exercise (e.g., resistance training and precise joint mobility training) and the psychological cognitive-behavioral elements of the relationship between the experience of pain-related fear have previously been reported to reduce work-related FAB in female workers with chronic musculoskeletal pain [18, 19]. Marchand and coworkers found that improvements in the work subscale of the fear avoidance questionnaire after cognitive-behavioral therapy and exercise predicted return to work within 12 months [20]. Similarly, reductions in the physical subscale of this questionnaire predicted disability at 12 months. These results highlight the relevance of being physically active to prevent disability and thus sickness absence. Further, Godges et al. [21] found that education and pain management counselling, physical activity, and exercise can reduce the number sick days in people with high fear avoidance beliefs and acute low back pain compared with standard physical therapy [21]. In addition, Frederiksen et al. [22] recently found in a cluster-randomized controlled trial that reassuring information delivered in a nonthreatening manner, about low back pain significantly, increased the odds for work participation and work ability among workers who experienced low back pain in a 12-month period [22]. These findings fit well with the biopsychosocial model first proposed by Engel [3] explaining the interconnected influence of biomedical, psychological, and social aspects of behavior in pain [3, 23]. Finding viable solutions to the immense socioeconomics costs of LTSA is a complex task as it is influenced by biomedical, psychological, and social factors and it is unlikely that any single intervention strategy will solve the problem. However, there is congruency among several studies that a high physical workload is associated with a significantly increased risk of developing work-related musculoskeletal disorders and LTSA [24–27], which should make future intervention strategies focus not only on physical activity but also include cognitive-behavioral elements to address workers' beliefs about work-related physical activity. As implementing changes in the specific working conditions (e.g. avoidance of monotonous repetitive, or heavy physical work) may be difficult or even impossible, intervention strategies focusing on the cognitive- and behavioral elements of what leads to pain reinforcement (e.g. pain catastrophizing) and subsequent FAB may contribute to a solution [28, 29]. National campaigns may also be a way forward to improve negative beliefs in relation to movement and musculoskeletal pain in the population [30, 31].

### 4.1. Strengths and Limitations

The present study has both strengths and limitations. Only two questions from the FABQ are included in the Danish Work Environment Cohort Study. Nevertheless, the prospective association with LTSA in the present study shows that these questions are relevant to consider in the prevention of LTSA. Another limitation is that there may be some circularity in the reasoning since the items used in the present study directly tap into physical activity and work. These items might correlate highly with self-rated “expectations” of working in the future which are not based on the fear avoidance model. Future studies should determine to what extent the full scale FABQ predicts LTSA. Further, only 53% replied to the questionnaire which could constitute a limitation. Nevertheless, a representative sample of more than 8000 workers with musculoskeletal pain from the general working population replying to the DWECS questionnaire is a large enough population to also constitute a strength of the present study. The use of register-based data on LTSA from DREAM further amplifies its strength. The validity of the DREAM register is high as the incentive for employers to report sickness absence allows them to seek compensation of employee sickness absence costs after 30 days of absence due to sickness thus reducing employer expenses. Finally, the use of sickness absence register data eliminates recall or reporting biases.

In conclusion, a very high level of fear avoidance is a risk factor for long-term sickness absence among workers with musculoskeletal pain regardless of the level of occupational physical activity. Future interventions should target fear avoidance beliefs through information and campaigns about the benefits of staying active when having musculoskeletal pain.

## Figures and Tables

**Table 1:** Demographics, health, and work-related characteristics of the study population.

	All			Sedentary workers			Physically active workers		
	N	Mean (SD)	%	N	Mean (SD)	%	N	Mean (SD)	%
Age, years	8319	43.7 (11.4)		3753	44.3 (10.8)		4349	43.2 (11.8)	
BMI (kg.m-2)	8173	25.4 (4.4)		3698	25.3 (4.5)		4265	25.6 (4.4)	
Gender									
Women	4779		57.5	2188		58.3	2469		56.8
Men	3540		42.6	1565		41.7	1880		43.2
Smoking									
No, never	3853		46.9	1904		51.2	1868		43.6
Ex-smoker	2417		29.4	1138		30.6	1228		28.6
Yes	1950		23.7	679		18.3	1192		27.8
Physical activity, leisure									
Low	1121		13.6	458		12.3	623		14.5
Moderate	5631		68.5	2680		72.0	2826		65.9
High	1467		17.9	585		15.7	839		19.6
Physical activity at work									
Sedentary work	3753		46.3	3753		100.0	0		0.0
Physically active work	4349		53.7	0		0.0	4349		100.0
Psychosocial work factors (0-100)									
Emotional demands	8161	45.3 (25)		3707	43 (23.3)		4287	47.2 (26.2)	
Influence at work	8100	66.6 (24)		3689	71 (22.4)		4246	62.9 (24.6)	
Support from colleagues	7651	72.5 (21.5)		3425	72.2 (21)		4072	72.8 (21.8)	
Support from leader	7822	68.6 (26)		3524	69.3 (25.8)		4141	68.2 (26)	
Pain intensity (0-9)									
Neck/shoulder	8301	3 (2.3)		3746	2.9 (2.2)		4339	3 (2.3)	
Low back	8310	2.9 (2.5)		3753	2.5 (2.4)		4341	3.2 (2.5)	
Arm/hand	8302	1.8 (2.3)		3747	1.6 (2.2)		4340	1.9 (2.4)	
Back disease									
No	6699		81.0	3099		82.9	3434		79.6
Yes	1568		19.0	641		17.1	879		20.4
Previous long-term sickness absence									
No	7537		90.6	3500		93.3	3844		88.4
Yes	782		9.4	253		6.7	505		11.6
Long-term sickness absence during 2-year follow-up									
No	7475		89.9	3450		91.9	3837		88.2
Yes	844		10.2	303		8.1	512		11.8

**Table 2:** Hazard ratios (HR) and 95% confidence intervals for long-term sickness absence during the 2-year follow-up in relation to fear avoidance beliefs (FAB) at baseline.

		N	%	Model 1	Model 2
				HR (95% CI)	HR (95% CI)
All	Very low FAB	1822	22.6	0.76 (0.62 - 0.93)	1.02 (0.80 - 1.29)
	Low FAB	1940	24.1	0.86 (0.71 - 1.05)	0.92 (0.73 - 1.16)
	Moderate FAB	2497	31.0	1	1
	High FAB	1042	12.9	1.31 (1.06 - 1.62)	1.13 (0.88 - 1.46)
	Very high FAB	761	9.4	1.80 (1.46 - 2.23)	1.48 (1.15 - 1.90)

Sedentary workers	Very low FAB	1049	28.8	0.70 (0.51 - 0.96)	0.86 (0.59 - 1.25)
	Low FAB	987	27.1	0.85 (0.62 - 1.16)	0.86 (0.60 - 1.23)
	Moderate FAB	1079	29.7	1	1
	High FAB	338	9.3	1.41 (0.98 - 2.04)	1.40 (0.91 - 2.15)
	Very high FAB	184	5.1	1.68 (1.08 - 2.61)	1.72 (1.04 - 2.83)

Physically active workers	Very low FAB	753	17.8	0.90 (0.68 - 1.18)	1.23 (0.89 - 1.70)
	Low FAB	925	21.9	0.91 (0.70 - 1.18)	1.00 (0.73 - 1.36)
	Moderate FAB	1344	31.9	1	1
	High FAB	660	15.6	1.21 (0.93 - 1.58)	1.08 (0.79 - 1.48)
	Very high FAB	538	12.8	1.69 (1.31 - 2.18)	1.48 (1.10 - 2.01)

Model 1: Adjusted for age and gender.

Model 2: model 1 + job group, psychosocial work environment (influence at work, emotional demands, support from colleagues, and support from leader), lifestyle (smoking, leisure physical activity, and BMI), musculoskeletal pain intensity (low back, neck/shoulder, and arm/hand), previous long-term sickness absence, mental health, and chronic disease (depression, diabetes, cardiovascular disease, cancer, and back disease).

## Data Availability

Due to the requirements for anonymity of participants in research projects in Denmark, it has not yet been decided how to share the data. People interested in performing analysis on the data should contact the research leader, Professor Lars L. Andersen, LLA@NRCWE.DK.
